# Desmopressin dose requirements in patients with permanent arginine vasopressin deficiency: a tertiary center experience

**DOI:** 10.1007/s11102-024-01454-4

**Published:** 2024-09-12

**Authors:** Emanuele Varaldo, Michela Sibilla, Nunzia Prencipe, Alessandro Maria Berton, Daniela Cuboni, Luigi Simone Aversa, Francesca Mocellini, Fabio Bioletto, Ezio Ghigo, Valentina Gasco, Silvia Grottoli

**Affiliations:** Division of Endocrinology, Diabetology and Metabolism, Department of Medical Sciences, University of Turin, Corso Dogliotti, 14, 10126 Turin, Italy

**Keywords:** dDAVP, AVP, Sublingual, Nasal, Central diabetes insipidus

## Abstract

**Purpose:**

The desmopressin daily dose requirement is highly variable among patients with arginine vasopressin (AVP) deficiency (i.e. central diabetes insipidus) and few studies to date have evaluated this topic, with often inconclusive results. The aim of our study was to identify clinical and biochemical predictors of such dose requirements in a cohort of patients with a confirmed diagnosis of permanent AVP deficiency who have good and stable control under substitutive treatment.

**Methods:**

We retrospectively analyzed data of all patients with permanent AVP deficiency undergoing regular follow-up at our Division. Inclusion criteria were the presence of stable disease under therapy for at least 12 months and in good biochemical and clinical control. Patients with AVP deficiency who lacked intact thirst or had a disease duration of less than 12 months were excluded from the analysis.

**Results:**

Out of the 132 patients initially screened, 96 patients (M/F 44/52; age 51 [37–63] years) met the inclusion criteria. Patients on nasal spray therapy (n = 8) had a significantly longer disease duration (p = 0.002) than patients treated with oral lyophilizate (n = 88). In the bivariate analysis, considering only patients treated with the sublingual formulation, the drug dose was correlated positively with estimated glomerular filtration rate (eGFR) and weight (r = 0.410, p < 0.001; r = 0.224, p = 0.036, respectively) and negatively with age (r = – 0.433, p < 0.001). In the multivariate regression analysis taking into account age, weight, and eGFR, only age emerged as a significant predictor of the required sublingual desmopressin dose (β = – 1.426, p = 0.044).

**Conclusion:**

Our data suggest that patient age appears to be the primary factor associated with the daily sublingual desmopressin dose required to achieve adequate clinical and biochemical control in patients with permanent AVP deficiency.

## Introduction

Arginine vasopressin (AVP) deficiency, also referred to as central diabetes insipidus, is a hydro-electrolytic balance disorder resulting from an alteration in the production or secretion of the hypothalamic neuropeptide [1, 2]. This condition can be of genetic or acquired origin, often occurring after neurosurgery [3, 4].

AVP deficiency is considered a rare disease, with an estimated prevalence in the population of approximately 1 in 25,000 individuals, and it belongs to the polyuria-polydipsia syndrome, along with AVP resistance (nephrogenic diabetes insipidus) and primary polydipsia [1, 5]. This syndrome encompasses a group of disorders characterized by hypotonic polyuria (generally defined as urine output > 50 mL/kg/day), accompanied by excessive drinking (generally defined as fluid intake > 3L/day) [6].

Indeed, the deficiency of the hypothalamic neurohormone results in a reduction of free water reabsorption at the level of the renal collecting duct (mediated through interaction with the V2 receptor), with a tendency towards rapid dehydration and possible consequent hypernatremia and hypertonicity, unless fluids are immediately replaced [1, 3].

The treatment of AVP deficiency, therefore, relies on restoring fluid losses in cases of hypovolemia, as well as on the chronic administration of a synthetic analogue of the human peptide, known as 1-deamino-8-D-AVP (dDAVP) or desmopressin [7]. This medication is exclusively selective for the renal V2 receptor, lacking the other effects mediated by interaction with the V1a receptor, primarily expressed at the vascular level, and V1b receptor, localized on the corticotrope cells of the anterior pituitary [8].

Therapeutic monitoring of subjects with AVP deficiency entails periodic measurement of serum sodium (s-Na) levels, with the aim of maintaining electrolytes within the desired range, as well as alleviating the often debilitating symptoms associated with polyuria, primarily by addressing nocturia [7].

One of the potentially most severe side effects associated with chronic desmopressin therapy, indeed, is dilutional hyponatremia, as the synthetic hormone is unable to regulate finely in response to any excess water intake like the native peptide [9]. Consequently, a strategy proposed by several authors is that of desmopressin escape, suggesting to the patient to periodically omit or delay therapy intake until the reoccurrence of polyuria [7].

Chronic desmopressin therapy can be administered either orally (tablets), sublingually (oral lyophilizate), or intranasally (spray or drops), and the daily medication dose often varies among different patients. In this regard, few studies to date have evaluated the needs for dDAVP dose requirement in adult or pediatric subjects, with inconsistent findings [10–13]. In fact, while some studies have observed a modest correlation between either age or body mass index (BMI) and daily desmopressin dose [10, 12], this finding was not confirmed by all studies [11]. Similarly, the study by Pedersen et al. reported a higher dose in subjects with congenital forms compared to patients with acquired forms [10], whereas the exact opposite was reported by Almutlaq and colleagues [11].

Moreover, in this regard, it is important to consider that the available evidence in the literature has usually determined the required dDAVP daily dose based only on the current dose at the last follow-up, which does not always represent the patient's actual need from both a clinical and biochemical perspective.

All this considered, the aim of our study was to evaluate the desmopressin dose requirements in a large cohort of patients with a confirmed diagnosis of AVP deficiency, who have good and stable clinical and biochemical control, in order to identify predictors of such dose requirements.

## Materials and methods

We retrospectively analyzed data of all patients with permanent AVP deficiency undergoing regular follow-up at the Division of Endocrinology, Diabetology and Metabolism of the University Hospital “Città della Salute e della Scienza di Torino” (Turin, Italy).

The following data were assessed for each patient: sex, weight (kg), height (cm), BMI (kg/m^2^), current age and disease duration, etiology of AVP deficiency, desmopressin formulation and total daily dose (μg), reported daily water intake and diuresis (mL), concurrent pituitary deficiencies and their current status. Moreover, data regarding s-Na, serum potassium (s-K), blood glucose, plasma osmolality (p-Osm), urine osmolality (u-Osm), creatinine and estimated glomerular filtration rate (eGFR, calculated through the CKD-EPI [Chronic Kidney Disease Epidemiology Collaboration]) were collected from the latest follow-up records.

Inclusion criteria were the presence of stable disease under therapy for at least 12 months and in good biochemical (defined as normal s-Na levels, with no episodes of hypo- or hypernatremia or need for desmopressin dose adjustment in the previous year) and clinical (defined as normal drinking habits < 3L/day and absence of nocturia, with a normal fluid balance) control.

On the other hand, patients with either AVP resistance, or AVP deficiency who exhibited apparently inadequate biochemical and/or clinical control, and lacked intact thirst, or had a disease duration of less than 12 months were excluded from subsequent analysis.

AVP deficiency was diagnosed based on the results of a water deprivation test or the need to continue desmopressin therapy 12 months after pituitary neurosurgery [3, 14]. For calculation of the daily dose requirements, we converted the nasal doses to the equivalent sublingual doses using the conversion factors previously proposed [15].

The study was approved by the Local Ethics Committee (cod. 0040828) and was in accordance with the principles of the Declaration of Helsinki. Written informed consent was obtained from all study participants.

## Statistical analysis

Normally and non-normally distributed variables were expressed as mean and standard deviation (SD) or median and interquartile range (IQR), respectively, while categorical data were expressed as counts and percent. Normality was assessed using the Shapiro–Wilk test.

Differences between groups were evaluated by Student's *t* test for independent samples in the case of variables with normal distribution; to highlight the differences between the median values of non-normally distributed variables Mann–Whitney test was used when appropriate.

The chi-square test and the Fisher’s exact test were used to evaluate the association between binary variables, while the Spearman’s test was used to evaluate the correlation of continuous ones. Additionally, multiple regression analysis was performed to identify clinical and biochemical variables associated with desmopressin daily dose.

A cut-off of p value < 0.05 was considered as statistically significant. Statistical analysis was performed using MedCalcTM® (Statistical Software version 20.007, MedCalc Software Ltd, Ostend, Belgium).

## Results

Between January 2019 and December 2023, 132 patients with permanent AVP deficiency were evaluated at the outpatient clinic of our Division. Among them, 36 patients were subsequently excluded: 15 patients presented with hyponatremia and 2 patients with hypernatremia during the last 12 months; 6 patients did not show good clinical control; in 9 patients, the desmopressin dose had been modified in the last 12 months and in 4 cases, the diagnosis of the disease had been made less than one year earlier.

**Fig. 1:**
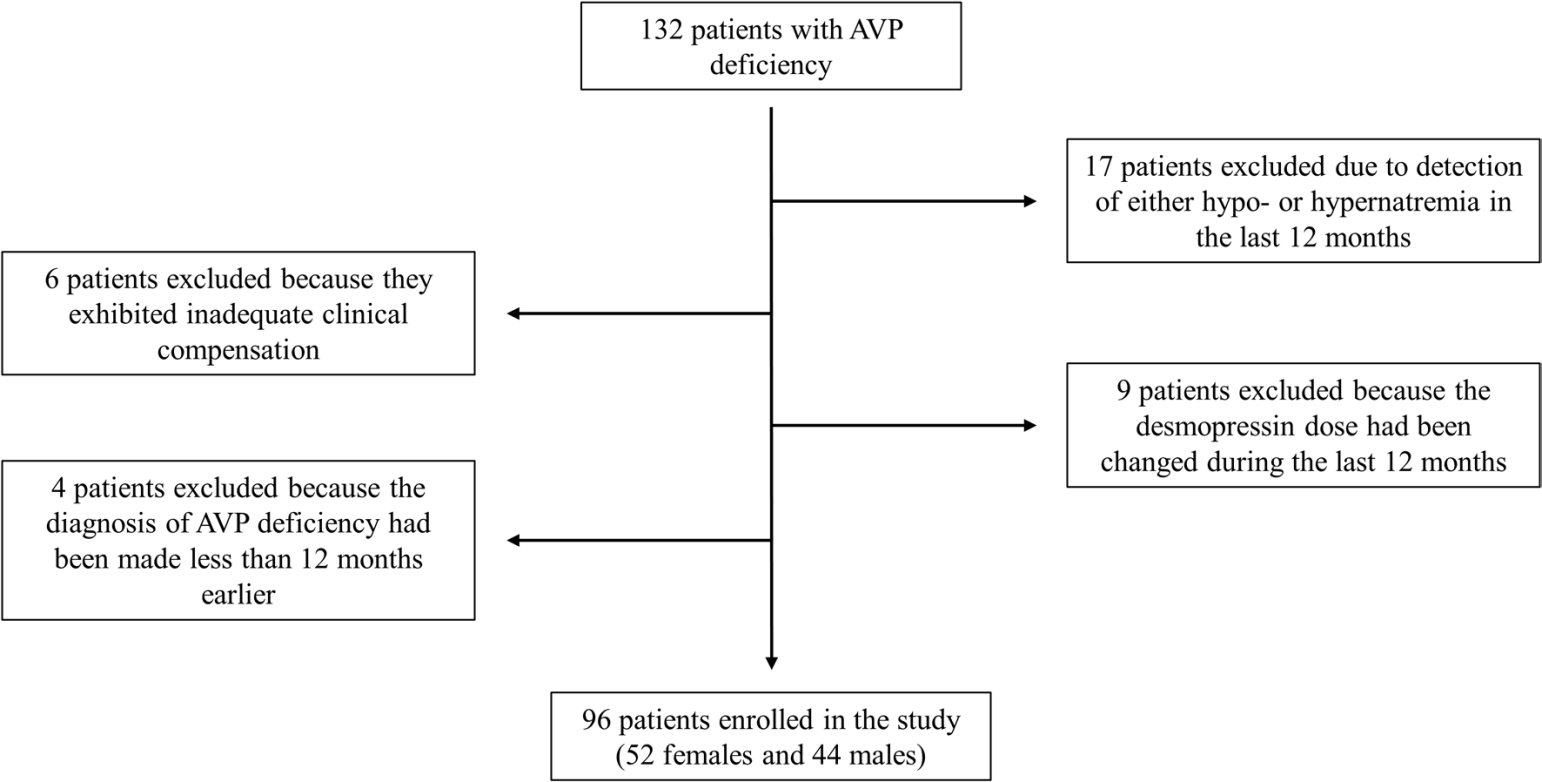
Enrollment process flowchart. *AVP* arginine vasopressin

Eventually, 96 patients (52 females and 44 males) were included in the study (Fig. 1).

## Patient characteristics

The anamnestic, general and biochemical characteristics of the patients enrolled in the study are presented in Table 1.

**Table 1:** Clinical and biochemical parameters of the patients. Data are expressed as mean ± standard deviation (SD) or median and interquartile range (IQR) or n (%)

Patient characteristics	Overall (n = 96)	Males (n = 44)	Females (n = 52)	p-value
Age, years	51 (37–63)	50 (39–61)	52 (35–64)	0.656
Height, cm	167 ± 9	174 ± 8	161 ± 6	**< 0.001**
Weight, kg	78.5 (64.0–88.8)	85.0 (76.5–96.0)	68.0 (59.5–80.0)	**< 0.001**
BMI, kg/m^2^	27.1 (24.5–31.1)	27.5 (25.6–31.5)	26.7 (23.1–30.5)	0.117
Obesity, n (%)	27 (28.1)	14 (31.8)	13 (25.0)	0.461
Diabetes mellitus, n (%)	12 (12.5)	8 (18.2)	4 (7.7)	0.136
Impaired renal function (defined as eGFR < 90 mL/min/1.73m^2^), n (%)	35 (36.5)	14 (31.8)	21 (40.4)	0.387
Growth hormone deficiency, *n (%)*	69 (71.9)	38 (86.4)	31 (59.6)	**0.004**
Central adrenal insufficiency, n (%)	72 (75.0)	39 (88.6)	33 (63.5)	**0.005**
Central hypothyroidism, n (%)	71 (74.0)	38 (86.4)	33 (63.5)	**0.011**
Hypogonadotropic hypogonadism, n (%)	65 (67.7)	37 (84.1)	28 (53.8)	**0.002**
At least one anterior pituitary deficit, n (%)	76 (79.2)	41 (93.2)	35 (67.3)	**0.020**
Anterior panhypopituitarism, n (%)	64 (66.7)	36 (81.8)	28 (73.1)	**0.004**
Previous neurosurgery, n (%)	66 (68.8)	35 (79.5)	31 (59.6)	**0.037**
Previous radiotherapy, n (%)	15 (15.6)	7 (15.9)	8 (15.4)	0.944
AVP deficiency etiology, n (%)				0.323
Non-functioning pituitary adenoma	7 (7.3)	3 (6.8)	4 (7.7)	
Secreting pituitary adenoma	5 (5.2)	3 (6.8)	2 (3.8)	
Craniopharyngioma	42 (43.7)	23 (52.3)	19 (36.6)	
Other sellar tumor	16 (16.7)	8 (18.2)	8 (15.4)	
Traumatic brain injury	6 (6.2)	3 (6.8)	3 (5.8)	
Idiopathic	9 (9.4)	1 (2.3)	8 (15.4)	
Hypophysitis	6 (6.2)	1 (2.3)	5 (9.6)	
Infiltrative disease	1 (1.1)	0 (0)	1 (1.9)	
Congenital	4 (4.2)	2 (4.5)	2 (3.8)	
Daily desmopressin dose, µg	120 (60–240)	120 (105–240)	120 (60–240)	0.532
Desmopressin formulation, n (%)				0.137
Sublingual	88 (91.7)	38 (86.4)	50 (96.2)	
Nasal	8 (8.3)	6 (13.6)	2 (3.8)	
Disease duration, years	13 (5–27)	16 (5–28)	13 (5–25)	0.825
s-Na, mmol/L	140 (139–142)	140 (139–141)	140 (139–143)	0.242
s-K, mmol/L	4.2 (3.9–4.4)	4.2 (3.9–4.3)	4.3 (4.0–4.6)	0.076
Blood glucose, mg/dL	83 (76–90)	84 (78–93)	82 (76–88)	0.159
p-Osm, mOsm/kg	285 (281–292)	285 (282–291)	285 (281–294)	0.524
u-Osm, mOsm/kg	564 (412–709)	590 (455–738)	541 (337–659)	0.068
Creatinine, mg/dL	0.85 ± 0.17	0.94 ± 0.16	0.77 ± 0.15	** < 0.001**
eGFR, mL/min/1.73m^2^	93 ± 19	98 ± 17	95 ± 20	0.528

The numbers in bold indicate significant values (*p* < 0.05)

*BMI* body mass index, *AVP* arginine vasopressin, *s-Na* serum sodium, *s-K* serum potassium, *p-Osm* plasma osmolality, *u-Osm* urine osmolality, *eGFR* estimated glomerular filtration rate (calculated through the CKD-EPI [Chronic Kidney Disease Epidemiology Collaboration])

The median age at the last visit was 51 (37–63) years, with a disease duration of 13 (5–27) years. The median fluid input in the whole cohort was 2000 (1500–2500) mL and was significantly lower in patients older than the median age of the population (2000 [1500–2063] vs 2000 [2000–2500] mL, p = 0.004).

The vast majority of patients had acquired AVP deficiency, while in only four patients the etiology was congenital (in two cases due to brain malformation and in two cases familial); similarly, most patients were on chronic therapy with the sublingual formulation of desmopressin, except for eight individuals receiving the nasal spray.

In the entire cohort, the daily dose of dDAVP was 120 (60–240; range 15–360) µg, with a dose of 120 (75–240) µg in patients using the sublingual formulation and an equivalent of 90 (45–150) µg in those using the nasal formulation (p = 0.129).

With respect to concomitant anterior pituitary function, only 24 out of 69 patients (34.8%) with growth hormone deficiency were on substitutive therapy, while all other patients with concomitant anterior pituitary hormone deficiencies were on replacement therapy and adequately controlled, except for women with secondary hypogonadism at an age compatible with physiological menopause (n = 11).

## Clinical characteristics and daily desmopressin dose

No difference in desmopressin dose was observed between the two sexes, either when analyzing the entire cohort (p = 0.532) or only patients on sublingual formulation (p = 0.393) (Table 1).

Patients on nasal spray therapy did not present significant differences in age compared to those on chronic therapy with the oral lyophilizate (p = 0.889), but they had a significantly longer disease duration (32 [25–39] vs 13 [5–22] years, p = 0.002). In fact, patients with the congenital form were more likely to use this formulation (p = 0.034).

On the other hand, no difference in the required dose of dDAVP was observed between patients with craniopharyngioma and those with other adenomatous and non-adenomatous tumors of the sellar region (p = 0.985). Similarly, no difference was evident in the drug dose between patients who previously underwent pituitary neurosurgery and those who did not (p = 0.994). Finally, no difference in the dose of dDAVP was observed between patients with anterior panhypopituitarism and those without (p = 0.242), as well as compared to those with preserved anterior pituitary function (n = 20, p = 0.225). Specifically, there was no difference in dDAVP dose between patients with and without central adrenal insufficiency (p = 0.199), even when stratified by sex (p = 0.701 for males, p = 0.140 for females).

In the bivariate analysis, there was a significant negative correlation between total daily desmopressin dose and age (r =  − 0.393, p < 0.001), which was only evident in patients treated with oral lyophilizate (r =  − 0.433, p < 0.001). On the other hand, no association was observed with disease duration (r = 0.094, p = 0.361 for the entire cohort, r = 0.144, p = 0.179 for patients treated with oral lyophilizate) (Table 2). Considering only patients on sublingual formulation, a modest correlation was observed between dose and weight (r = 0.224, p = 0.036), while no correlation was observed with BMI (r = 0.161, p = 0.135). However, obese patients required a higher dose compared to non-obese subjects (240 [120–240] vs 120 [60–180] µg, p = 0.007). Finally, no difference in desmopressin dose was observed between patients with and without diabetes mellitus, either analyzing the entire cohort (p = 0.140) or when stratified by sex (p = 0.244 for males, p = 0.337 for females).

**Table 2:** Association between clinical and biochemical parameters and sublingual desmopressin dose using the Spearman’s correlation coefficient (r) and the multivariate regression analysis (β)

Variable	Correlation (r)	p-value	Multivariate (β)	p-value
Age, years	− 0.433	** < 0.001**	− 1.426	**0.044**
Disease duration, years	0.144	0.179		
Height, cm	0.193	0.072		
Weight, kg	0.224	**0.036**	0.833	0.063
BMI, kg/m^2^	0.161	0.135		
Creatinine, mg/dL	− 0.180	0.094		
eGFR, mL/min/1.73m^2^	0.410	**< 0.001**	1.155	0.073

The numbers in bold indicate significant values (*p* < 0.05)

*BMI* body mass index, *eGFR* estimated glomerular filtration rate (calculated through the CKD-EPI [Chronic Kidney Disease Epidemiology Collaboration])

## Biochemical characteristics and daily desmopressin dose

In the bivariate analysis, there was a significant correlation between the daily desmopressin dose and eGFR values (r = 0.343, p = 0.001), which was only evident in patients treated with the oral lyophilizate formulation (r = 0.410, p < 0.001). Indeed, patients with even modestly impaired renal function (eGFR < 90 mL/min/m^2^, n = 35) required a lower daily dose (180 [120–240] vs 90 [60–143] µg, p < 0.001) and, as expected, were significantly older (61 [53–72] vs 45 [30–53] years, p < 0.001) compared to other subjects.

In the multivariate regression analysis taking into account age, weight, and eGFR, only age emerged as a significant predictor of the required sublingual dDAVP dose to achieve good clinical and biochemical control (β =  − 1.426, p = 0.044) (Table 2).

## Discussion

The results of our study highlight that in patients with permanent AVP deficiency treated with the sublingual desmopressin formulation, age is the main predictor of the drug dose required to achieve good clinical and biochemical control. In particular, our findings clearly confirm that there is a negative correlation between age and dDAVP dose requirement.

The data currently available in the literature on the variability of desmopressin dose requirements in patients with AVP deficiency are limited and are primarily represented for the oral or intranasal formulations [10–12], while evidence regarding desmopressin dose in patients using the sublingual formulation is scanty.

The sublingual formulation, similar to the nasal one, has an increased bioavailability compared to tablets, since orally delivered dDAVP is partly degraded by gastric acid [16].

In this regard only one Japanese paper has been recently published which identified sex, age and eGFR as significant factors associated with desmopressin dose requirements [13].

In our study, age was the only factor confirmed to be associated with the daily dose of dDAVP. The presence of age-related dysfunction of the hypothalamic-neurohypophyseal-renal axis, indeed, has been known for more than 60 years now [17, 18]. Several mechanisms have been proposed, including changes in hypothalamic-pituitary regulation of thirst and AVP secretion, as well as hypersensitivity of the osmoregulatory system [18, 19].

Older subjects often present with decreased thirst, likely as a consequence of a higher osmolar set point for thirst sensation [20]. In our cohort, indeed, subjects older than the median age of the population reported a lower fluid intake compared to younger subjects. Moreover, most of the literature regarding water homeostasis has demonstrated that older individuals exhibit a greater increase in AVP secretion per unit change in plasma osmolality than younger subjects [18, 19, 21], supporting the theory of an increase in osmoreceptor sensitivity with aging.

The underlying cause of the age-related increase in sensitivity of the osmoregulatory system has not been fully elucidated yet. It may partially be due to increased pituitary reserves of readily releasable AVP, as histological studies have shown an accumulation of neurosecretory material in the posterior pituitary of elderly individuals [19]. Alternatively, it could represent a compensatory response to the diminished baroreceptor-mediated control of AVP secretion in response to hemodynamic changes, which has been consistently documented in the elderly [18, 19, 22].

Finally, older individuals often exhibit an impaired capacity to excrete free water in the urine, as well as reduced renal function; in this regard, the eGFR decreases by approximately 1 mL/min/1.73m^2^ per year starting at the age of 40, with a more pronounced acceleration beginning at age 65 [18, 23]. In our population, in fact, patients with even slightly reduced renal function (eGFR < 90 ml/min/1.73m^2^) were significantly older than other subjects.

While eGFR showed a positive association with the desmopressin dose in the bivariate analysis, this significance was lost in the multivariate analysis. This modest correlation is not an unexpected finding: the pharmacokinetics of dDAVP involve a major proportion of excretion through the kidneys, and it has been demonstrated that the drug's half-life is longer in patients with impaired renal function [24, 25]. In our cohort indeed, patients with even mild reduction in eGFR required significant lower doses than those with normal renal function.

Taking all factors into account, it is plausible that both reduced renal function, with consequent diminished desmopressin clearance, and lower fluid intake observed in older subjects could contribute to the negative correlation with age evident in our study, which was also confirmed in the multivariate analysis. It is worth noting that although renal function specifically lost statistical significance in the multivariate analysis, the result remained borderline significant.

In contrast to the study by Hoshino et al., however, no difference in the daily desmopressin dose between males and females was observed. Several studies in the past have reported a greater susceptibility of women to desmopressin, with a risk up to 5 times higher of developing dilutional hyponatremia compared to males [26–28]. Nonetheless, these findings stem from studies conducted on both healthy subjects and individuals with nocturnal enuresis, while data in patients with AVP deficiency are currently lacking. In this regard, it is therefore unknown whether the increased susceptibility of females to desmopressin is also present in a condition of chronic hormonal deficiency.

Moreover, an important difference between our study and that of Hoshino et al. concerns the characteristics of the patients included in the analysis. While in their work, patients were recruited immediately post-pituitary neurosurgery and were evaluated for up to 12 months [13], in our study only patients with a disease duration of more than 12 months were considered.

The diagnosis of permanent postoperative AVP deficiency is generally made based on the persistence of the hydro-electrolytic disorder and the need to continue dDAVP therapy for more than 12 months after surgery, as the recovery of antidiuretic function after one year is considered exceptional [3]. In contrast, in the immediate post-surgery period, various disorders of AVP secretion can be present, such as non-pathological polyuria and transient AVP deficiency [3, 4, 29, 30].

Based on our results, it cannot be excluded that the previously reported greater susceptibility to dDAVP in women, if also present in patients with AVP deficiency, may reduce over time, resulting in a similar drug dose between the two sexes.

In our cohort, moreover, males had undergone neurosurgery more frequently than females and presented a higher incidence of anterior pituitary deficiency. However, none of these variables were associated with the dose of dDAVP required to achieve good clinical and biochemical control.

In previous studies, a positive association between BMI and desmopressin dose has also been observed, more evident for the tablets [10, 12], although a weak association has also been reported in patients using the orally lyophilized formulation [13]. In our study, a modest association was observed between drug dose and weight while a clear correlation with BMI was not evident; anyway, obese patients required higher doses compared to other subjects.

In this regard, the greatest evidence is for the oral drug, and this could depend on differences in the pharmacokinetics of parenteral formulations compared to tablets. Indeed, the latter must be absorbed in the digestive system before entering the bloodstream, while nasal and sublingual formulations are absorbed directly into the bloodstream through the mucosa [16].

Moreover, Pedersen et al. also observed a higher dose requirement in patients with AVP deficiency after removal of craniopharyngioma compared to patients undergoing neurosurgery for other pituitary tumors [10], while no difference was observed in our population.

Patients with craniopharyngioma are at very high risk of postoperative AVP deficiency because surgery is usually more invasive, often involving the pituitary stalk, and can sometimes result in complete loss of thirst sensation (adipsic AVP deficiency) [3, 31, 32].

In the aforementioned study, information regarding thirst sensation was not provided, and approximately 30% of patients had experienced at least one episode of hyponatremia in the previous 12 months [10]. It is therefore possible that the evidence of a higher dose of dDAVP in patients with craniopharyngioma was simultaneously affected by a higher prevalence of hyponatremia in this group of patients. It is important to note that previous studies have only considered the current dose of dDAVP at the last follow-up, which however does not always reflect the patient's actual needs.

In this regard, the definition of good control for AVP deficiency during desmopressin therapy is not straightforward, but in an attempt to be as homogeneous as possible, we included in our study only patients with a disease duration of at least 12 months and in good clinical and biochemical control without the need for dosage adjustments in the last year of therapy.

Finally, in contrast with the evidence from the studies by Pedersen et al. and by Almutlaq et al. [10, 11], we did not observe any difference in the dDAVP dose among patients with congenital or acquired etiology. However, only a very small proportion of patients were affected by the congenital form in our cohort. Of note, those patients were significantly more likely to be treated with nasal desmopressin and, as expected, presented a longer duration of disease.

Regarding nasal desmopressin formulation, several studies in the past have reported a higher risk of hyponatremia compared to the oral formulation [33, 34]. At the same time, this drug exhibits non-constant absorption, as inflammatory processes at the level of the nasal mucosa can interfere significantly [35].

Then, it should not be surprising that the majority of patients in our study used the sublingual formulation, and mostly patients with a very long disease duration were on chronic therapy with nasal desmopressin, given that sublingual desmopressin has only been available since 2005 [16, 33].

Our study presents some strengths and limitations. One major strength is the rigorous inclusion of only patients with permanent AVP deficiency with intact thirst sensation and in good biochemical and clinical control under stable dose of substitution therapy for at least 12 months. We have thus reasonably avoided the inclusion of transient postoperative forms and have used clinically and biochemically recognized parameters as fundamental elements to define the appropriate drug dose for each patient.

The main limitations of the study include its retrospective design and the fact that a confirmation test was available only for a smaller proportion of patients, considering that the etiology of the disorder was mostly post-neurosurgical. Anyway as suggested by several experts, patients were correctly instructed to periodically omit or delay desmopressin intake until the reoccurrence of polyuria, which occurred in all cases [7].

## Conclusion

Based on our data, patient age appears to be the primary factor associated with the daily sublingual desmopressin dose required to achieve adequate clinical and biochemical control in patients with permanent AVP deficiency.

Further studies involving larger cohorts of patients are necessary to clarify whether a greater susceptibility to dDAVP is also present in women with AVP deficiency and whether, as proposed, this susceptibility may diminish over time in the context of chronic hormonal deficiency.

## Data Availability

The data sets generated during and/or analyzed during the current study are not publicly available but are available from the corresponding author on reasonable request.

## Declarations

### Research involving human and animals rights

All procedures performed in studies involving human participants were in accordance with the ethical standards of the institutional and/or national research committee and with the 1964 Helsinki declaration and its later amendments or comparable ethical standards.
